# Influence of Retinal Microsecond Pulse Laser Treatment in Central Serous Chorioretinopathy: A Short-Term Optical Coherence Tomography Angiography Study

**DOI:** 10.3390/jcm10112418

**Published:** 2021-05-29

**Authors:** Michelle Prasuhn, Yoko Miura, Aysegül Tura, Felix Rommel, Vinodh Kakkassery, Svenja Sonntag, Salvatore Grisanti, Mahdy Ranjbar

**Affiliations:** 1 Department of Ophthalmology, University Hospital Schleswig-Holstein, University of Lübeck, Ratzeburger Allee 160, 23538 Lübeck, Germany; Ayseguel.Tura@uksh.de (A.T.); felix.rommel@uksh.de (F.R.); Vinodh.Kakkassery@uksh.de (V.K.); svenja.sonntag@uksh.de (S.S.); salvatore.grisanti@uksh.de (S.G.); eye.research101@gmail.com (M.R.); 2 Laboratory for Angiogenesis & Ocular Cell Transplantation, University Hospital Schleswig-Holstein, University of Lübeck, Ratzeburger Allee 160, 23538 Lübeck, Germany; 3 Institute of Biomedical Optics, University of Lübeck, Peter-Monnik-Weg 4, 23562 Lübeck, Germany

**Keywords:** OCTA, central serous chorioretinopathy, choroidal perfusion, choriocapillaris, Sattler’s layer, Haller’s layer, retinal perfusion, subthreshold micropulse laser

## Abstract

Background: Central serous chorioretinopathy (CSC) is a common macular condition characterized by detachment of the neuroretina and is a frequent cause of central vision loss in adults. Among the various therapeutic strategies, subthreshold microsecond pulsed laser (SML) treatment has become a useful option. Despite the suggested involvement of choroidal circulatory disturbances in CSC, the effects of this treatment on macular microperfusion have not been fully evaluated yet. Herein, we report the impact of SML on retinal and choroidal microvascular flow using non-invasive optical coherence tomography (OCT) angiography (OCTA). Methods: In this study, CSC patients with persistent subretinal fluid (SRF) with or without secondary choroidal neovascularization (CNV) were included (referred to as the pachychoroid neovasculopathy (PNV) group and the CSC group, respectively). SML was conducted using a yellow (577 nm) laser with a duty cycle of 10%, spot size of 200 µm and duration of 200 ms. Best corrected visual acuity (BCVA) as well as OCT and OCTA images were evaluated at baseline and 4 weeks after SML. OCTA parameters of interest included full retinal perfusion (FRP), choriocapillaris perfusion (CCP), Sattler’s layer perfusion (SLP), and Haller’s layer perfusion (HLP), which were evaluated longitudinally and compared to unaffected fellow eyes. Results: 27 affected eyes and 17 fellow eyes from 27 patients were included. Before treatment, central retinal thickness (CRT) and subfoveal choroidal thickness (SFCT) of affected eyes were significantly larger than in fellow eyes. Four weeks after SML, CRT decreased significantly, whereas perfusion parameters did not change. In subgroup analyses, the CSC group showed a significant decrease in SFCT, whereas the PNV group did not despite the decrease in CRT. Conclusion: Our results suggest that the SML may affect the SFCT of the CSC, but not the PNV patients at least within four weeks following treatment. This effect seems to be independent of the change in choroidal perfusion measured with OCTA.

## 1. Introduction

Central serous chorioretinopathy (CSC) is a common chorioretinal disease and a frequent cause of central vision loss, primarily affecting people between 20 to 60 years of age. It is characterized by serous detachment of the neuroretina, typically with localized disruption of the outer blood retinal barrier, where the tight junctions of the retinal pigment epithelium (RPE) are localized [1]. CSC is an entity of the pachychoroid disease spectrum, in which the choroidal vessels are typically dilated, and leakage might be seen in indocyanine green angiography (ICGA) [2,3]. This mechanism is suggested to lead to choriocapillaris hypoperfusion, consequently causing localized RPE damage [4]. Conditions of the pachychoroid disease spectrum are characterized by a focal or diffuse increase in choroidal thickness. Specific findings include dilated vessels in the Haller’s layer, the so called pachyvessels. The choroidal substructures are altered and a shift in thickness as well as perfusion from the choriocapillaris (CC) and Sattler’s layer (SL) towards Haller’s layer (HL) can be seen [5]. CSC patients may develop choroidal neovascularizations (CNVs), which have recently been termed pachychoroid neovasculopathy (PNV) and is also part of the pachychoroid disease spectrum [6]. 

Patients often complain of substantial visual impairment, which consequently affects their quality of life. Treatment for CSC aims to preserve outer sensory retina layers and achieve quick and complete resolution of SRF. Because of the frequently self-limiting course, treatment modalities should have a favorable safety profile. 

Photocoagulation at RPE leakage points is a traditional evidence-based method to treat CSC. However, it has several serious drawbacks, including symptomatic scotomas, foveal distortion, and subretinal fibrosis, and thus has to be avoided in cases with subfoveal to juxtafoveal leaking points [7]. Enlargement of the laser spot may also threaten central vision. In the last decades, further treatment options have been introduced: diuretics, half-dose photodynamic therapy (PDT), minimally-invasive laser therapy such as selective retina therapy (SRT), and treatment with a microsecond pulsed laser. One of the diuretics, eplerenone, is a steroidal antimineralocorticoid and has been used widely with CSC patients in recent years [8]. Although its potential effect on the choroidal blood flow has been suggested [9], recent study results showed no significant therapeutic effect of eplerenone in chronic CSC patients [10,11]. Half-dose PDT is a safe and effective method to treat juxtafoveal or subfoveal leakage [9]. However, long-term adverse effects or cumulative effects of repeated treatments, such as RPE atrophy, still cannot be excluded and thus caution should be exerted [12] 

Subthreshold microsecond pulsed laser (SML) treatment has also been proposed as a new therapeutic strategy in CSC. This is a method to reduce the total energy of the irradiated laser pulse by reducing the on-time during one irradiation. It may easily achieve a very mild increase in temperature of RPE cells such that cells are sublethally heated or just mildly coagulated. Sublethal RPE hyperthermia has been shown to activate different intracellular molecules, such as heat shock protein 70 [13,14,15] and water transport protein aquaporine [16]. Furthermore, it has been shown to increase the antioxidative potential [17,18] and to reduce the thickness of the Bruch’s membrane in an age-related macular degeneration (AMD) mice model [19,20,21,22]. Thus, sublethal RPE thermal treatment gained increasing attention as a new therapeutic option for different macular disorders, such as CSC, diabetic macular edema and AMD [20,23]. In particular, the PLACE trial and a recent meta-analysis demonstrated that SML may be considered as a competitive alternative to PDT for treating chronic CSC, and as the first-line treatment of chronic CSC [24,25].

Although there is increasing clinical evidence for the treatment of CSC using SML [20], the effect of the treatment on the choroid has not been thoroughly investigated to date. Therefore, in the present study, we aimed to investigate structural and functional alterations of the retina and the choroidal sublayers in patients with CSC before and after SML using OCT and OCTA.

## 2. Materials and Methods

### 2.1. Study Participants

The participants for this prospective observational study were enrolled at the Department of Ophthalmology of the University of Lübeck. The study was positively reviewed by the institutional review board and adhered to the tenets of the Declaration of Helsinki (ID 18-102). All participants received detailed information about the study, and written informed consent was obtained individually by each participant before enrolment. We included patients who were diagnosed with CSC based on the SRF involving the macula as demonstrated by spectral domain optic coherence tomography (SD-OCT) and persistent SRF for at least 3 months. Both local and diffuse leakage on FA were included in this study. Exclusion criteria were: (1) history of any other macular disease; (2) any laser treatment within six months before enrolment (3); intravitreal injection within three months of enrolment; (4) large RPE atrophy; (5) history of ocular diseases other than CSC in the fellow eye; (6) previous PDT. Patients who were treated with diuretics, such as acetazolamide and eplerenone, were included in the study. As control, we examined the fellow eyes, which received the same multimodal imaging modalities. If the fellow eyes had a history of previous retinal disease, including active or inactive CSC, or showed signs of CSC in ICGA, OCTA, or OCT these eyes were excluded from the analysis. For subgroup analyses, we differentiated between CSC patients without CNV and PNV patients. The latter was characterized by the presence of a CNV, which was diagnosed using FA, OCT, ICGA, and OCTA by two experienced ophthalmologists in a consensus process (Y.M. and M.P.).

### 2.2. Study Protocol

At baseline, all participants underwent a thorough examination including refraction, best-corrected visual acuity (BCVA) in Snellen, intraocular pressure (IOP), slit-lamp biomicroscopy, macular enhanced-depth imaging (EDI) OCT, FA, ICGA, FAF as well as OCTA. The maximum permissible spherical and cylindrical aberration was ±3 and ±1 diopters, respectively. Imaging was performed using the Zeiss Cirrus HD-OCT (AngioPlex, CIRRUS HD-OCT model 5000, Carl Zeiss Meditec, Inc., Dublin, CA, USA) by a single, trained operator. Each imaging session included EDI-OCT (10 × 10 mm²) and OCTA (6 × 6 mm²) volumetric scans of the posterior pole. Only scans with a signal strength ≥7, centered on the fovea, and without motion-, segmentation-, and projection artifacts were included [26]. To avoid inaccuracies due to diurnal changes in perfusion, OCTA images were acquired at the same 2 h time frame of the day [27,28]. One month after treatment, all participants underwent the same examination of both eyes, however without FA. 

### 2.3. Laser Treatment

Laser irradiation was conducted using a yellow laser (577 nm) with a system for microsecond pulsing (MicroPulse TxCell Scanning Laser Delivery System; Iridex, Mountain View, CA, USA or Truscan; LIGHTMED, San Clemente, CA, USA), with a spot size of 200 µm and a duration of 200 ms with a 10% duty cycle. Test irradiation was conducted outside of the upper or lower vascular arcade, starting from a power of 200 mW, with increments of 100 mW, until a mild white spot was observed (determination of the visibility threshold). Treatment was conducted with the power of 40–50% of the visibility threshold around the leakage points observed in FA. Irradiation was conducted with a spacing about 1 spot diameter. In cases where there was no leakage point or diffuse leakage, SML was applied diffusely at the area of SRF. 

### 2.4. OCT and OCTA Data Analysis

Central retinal thickness (CRT) was automatically measured by the device, as defined by the Early Treatment Diabetic Retinopathy Study (ETDRS) in the OCT scans [29]. The total macular volume (TMV) was also automatically calculated. Subfoveal choroidal thickness (SFCT) was measured manually in EDI-OCT scans just below the fovea, extending perpendicularly from the hyperreflective Bruch’s membrane layer to the inner scleral border. 

**Figure 1:**
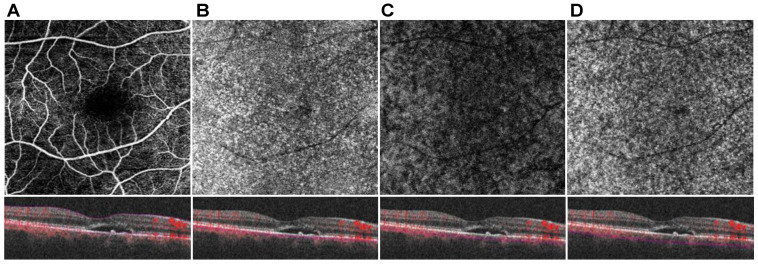
Optical coherence tomography angiography (OCTA)-Imaging of the posterior pole in a subject with Central Serous Chorioretinopathy. En face angiogram and corresponding B-scan at the level of the full retina (**A**), choriocapillaris (**B**), Sattler’s layer (**C**), and Haller’s layer (**D**).

**Figure 2:**
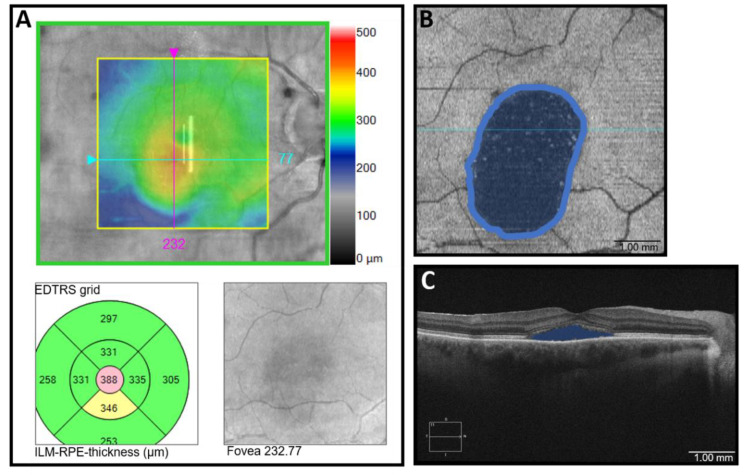
Visualization of methods in optical coherence tomography (OCT) and OCT- angiography in a patient with Central Serous Chorioretinopathy (**A**): Determining lesional retinal thickness = LRT: En face Analysis. The top panel shows an image of a retinal thickness map. The thickest point with the maximum extent of subretinal fluid (SRF) was determined and fixed, and the EDTRS grid was centered on this point (bottom left panel) to determine the retinal thickness (388 µm). Inner limiting membrane = ILM; Retinal pigment epithelium = RPE. (**B**): Measuring SRF area: Inner segment/outer segment ellipsoid analysis pictures of the base area of the SRF spot. The area was measured in ImageJ. (**C**): Measuring apical SRF = aSRF: OCT B-scan. The maximum extent of SRF was measured using the device’s measuring tool.

Figure 1 shows an overview of OCTA segmentation. Figure 2 visualizes the OCT analyses to characterize the extent of the SRF. The maximum point of the SRF was determined and the focal retinal thickness was automatically measured using the ETDRS grid (lesional retinal thickness = LRT). Additionally, in the B-scan with the maximum SRF, the area of SRF was measured using the device’s measuring tool (apical SRF = aSRF). We also assessed the SRF base area, which was pictured in the inner segment/outer segment images of the device and was calculated using the area measuring tool in ImageJ ((NIH, Version 1.48b, Bethesda, MD, USA). Manual measurements were performed by two experienced graders (F.R. and M.P.) who were blinded to the clinical information about the examined eyes and the results were averaged. 

OCTA data were manually segmented in all B-Scans to achieve retinal and choroidal sublayer slabs. Slabs for full retina perfusion (FRP) were measured from the Bruch’s membrane to the inner limiting membrane (ILM). The choroidal sublayers were manually segmented to get representative 20 µm slabs of the CC, SL, and HL according to previously published protocols [26,27]. All acquired en face images were exported into ImageJ for thresholding. Binarization was performed using the Otsu method, which is an automatic threshold selection from grey-level histograms [30]. As suggested by Nicolò et al., CC perfusion (CCP) was calculated by scoring the percentage of white pixels, while for SL perfusion (SLP) and HL perfusion (HLP) the values of black pixels were taken into account [31]. As previously mentioned, a feature of pachychoroidal diseases is a perfusion shift from CC and SL towards HL. To calculate the specific shift of perfusion between the choroidal sublayers, ratios between CCP, SLP, and HLP were determined (CCP/HLP, and SLP/HLP). To analyze the perfusion metrics in the pathological area, a circle of 1000 µm diameter was set around the maximum extent of SRF and perfusion values were calculated for CC, SL, HL, FR as described above using the Otsu method for binarization (=lesional CCP, lesional HLP, lesional SLP). The specific perfusion shifts were also calculated below the encircled lesion (=lesional CCP/HLP and lesional SLP/HLP) as described above.

### 2.5. Statistical Analysis

Exploratory data analysis was calculated using IBM SPSS (Version 24.0, Chicago, IL, USA) and figures were generated using Prism GraphPad (Version 8.0, La Jolla, CA, USA). Snellen BCVA was converted to the logarithm of the minimum angle of resolution (logMAR). Quantitative variables were summarized as median and Quartiles (Q1 and Q3) and qualitative variables as frequency and percentage. The Shapiro–Wilk test was used to check for normality of the obtained data. Baseline and follow-up values of the same eye were compared using a Wilcoxon signed rank test. Differences between CSC eyes and fellow eyes were assessed by a Mann–Whitney U test. For all tests, values of *p* < 0.05 were considered statistically significant. 

## 3. Results

Twenty-seven CSC patients were considered eligible and were included in this study. Seventeen unaffected fellow eyes served as a control. The demographic features of these groups are listed in Table 1. The anterior segment examinations did not reveal any relevant features. The biomicroscopic fundus examinations revealed serous retinal detachment at the posterior pole, particularly in the macular region, and multiple zones of RPE alterations (hypo- as well as hyper-pigmentations and atrophic changes). Neither drusen nor subretinal hemorrhages were detected.

Table 2 summarizes the anatomical and functional parameters of the CSC and fellow eyes. Eyes with CSC showed a significantly worse BCVA with an increased CRT and SFCT. Perfusion values of the retina and choroidal sublayers did not differ significantly.

Anatomical and functional parameters at baseline and follow-up are presented in Table 3. CRT decreased significantly after SML (from 306 µm to 266 µs, *p* < 0.05). The median value of the LRT decreased significantly after SML. We did not detect any significant changes in other parameters, including BCVA, SFCT, SRF and perfusion values. 

Table 4 comprares demographic parameters of our subgroups: patients with CSC and PNV.

Table 5 lists anatomical and functional parameters in the two subgroups at baseline and four weeks after SML. Significant reductions were identified in the CRT of the PNV group (from 325 µm to 267 µm, *p* < 0.01) and the SFCT of the group of CSC patients without CNV (from 329 µm to 299 µm, *p* < 0.05). 

Table 6 shows the statistical results of the comparison of all parameters between two subgroups, CSC without CNV and PNV, before and four weeks after treatment. The patients with PNV showed worse BCVA at both time points, but there was no difference in other parameters in OCT and OCTA. 

## 4. Discussion

In this prospective OCTA-based study, we investigated retinal and choroidal vascular alterations in patients diagnosed with CSC before and four weeks after SML. To the best of our knowledge, this is the first study to evaluate the impact of SML on choroidal sublayer perfusion in CSC patients in detail. Our main aim was to assess vascular changes of substructure perfusion due to SML in a short-term follow-up period. To gain deeper insights into the characteristics of conditions of the pachychoroid disease spectrum, we differentiated between CSC and PNV patients and found a different response to SML in these subgroups. The pathophysiology of CSC and other pachychoroid conditions is not fully understood, so many study groups focus on in vitro and in vivo studies. 

It is known that there is an imbalance of oncotic and hydrostatic pressure in CSC due to multiple factors. Growing evidence indicates that the pathomechanisms of CSC are associated with dysfunction of the thickened choroid with subsequent impairment of the RPE. Some authors have postulated that downregulation of the cell–cell adhesion molecules in the vascular endothelium could increase the permeability of choroidal vessels, causing fluid leakage under the neurosensory retina [32]. Our study aimed to add to previous information about pathophysiological and clinical characteristics. CSC affects men more than women and usually occurs between the second and sixth life decade. Our epidemiological data concerning age and gender correspond to other studies on CSC patients [33,34]. Predictably, BCVA in our CSC cohort was significantly worse compared to unaffected eyes (*p* < 0.001), as most of the patients in this study suffered from persistent CSC with a relatively strong visual impairment and needed further intervention. Since all patients had substantial SRF before SML, CRT was significantly thicker in affected eyes than in fellow eyes (*p* = 0.047). SML has been introduced as a therapy modality for CSC with persistent fluid. For our study, we included only patients who showed SRF for at least 3 months and demonstrated typical findings of active CSC on multimodal imaging; thus, SRF would not be expected to resolve spontaneously in these patients. BCVA improved over the follow-up period (0.4 vs. 0.3 logMAR; *p* = 0.348), even though not statistically significant. We noticed a significant reduction in CRT after SML (306 vs. 266 µm; *p* = 0.024). However, the BCVA and CRT outcome after SML usually change most over a longer period, which is not covered by our study design [7,20,25].

Subretinal fluid parameters decreased noticeably over the follow-up period without reaching statistical significance (*p* = 0.115 for aSRF and *p* = 0.123 for SRF area). The retinal thickness around the SRF lesion decreased significantly (364 vs. 333 µm; *p* = 0.044). It has already been shown that the morphological changes and reduction in subretinal fluid do not always correspond to the functional outcome and can be delayed [25]. However, resolution of SRF is a prerequisite for preserving or restoring function and thus the increase in the BCVA might be noticeable at later follow-up points.

As a condition of the pachychoroid disease complex, our CSC cohort had a significantly thicker SFCT than the unaffected fellow eyes (*p* = 0.022). Pachychoroid disease is characterized by the attenuation of the choriocapillaris overlying dilated choroidal veins with consecutive alteration of the RPE and retina [5]. The choroid is usually focally or diffusely thickened, which can also be seen in our measurements of the CSC group. Fellow eyes of CSC patients tend to have a thicker choroid even if they do not show any clinically significant pathologies [35]. The median choroidal thickness in our control group was 275 µm, which is within a normal range. However, while a thick choroid is frequently seen, choroidal thickness per se is not the most important criterion for defining the pachychoroid disease phenotype. The term pachychoroid covers several, also substructural morphological features that usually affect both eyes, even though to a different extent. It has already been reported that persistent structural changes can be detected even in unaffected fellow eyes of CSC patients [36]. Therefore, even if the unaffected fellow eyes of our control group showed no obvious pathological alterations concerning RPE and the retina, the choroid and choroidal substructures might still be altered in comparison to healthy patients. Ho et al. also investigated the changes of the choroidal thickness after SML in CSC patients [37]. They used a choroidal EDTRS grid to detect focal and volume changes which revealed no effect over their short-term and long-term follow-up period. They also analyzed differences in perfusion before and after SML compared to PDT in CSC patients and showed a significant change in perfusion in central 3x3 mm angiograms of the choriocapillaris after six months but not after four weeks. We aimed to provide further detailed analyses that focus specifically on the changes in vascular sublayers to gain a deeper insight into microvascular perfusion changes. Additionally, 6 × 6 mm angiograms in the current study might lead to a better overview of perfusion changes not only just around the fovea. OCTA delivers highly detailed, three-dimensional images of the microvasculature of the retina and choroid and helps to assess retinal perfusion. Several groups have already reported key findings regarding OCTA in CSC patients compared to healthy controls [38,39]. In CSC patients, the foveal architecture is distorted, and slab segmentation can be difficult. Primarily, the inner plexiform layer (IPL) is prone to inaccurate segmentation and occurs in 64.3%, whereas there are almost no errors concerning segmentation of the RPE or ILM [40]. However, the IPL is the most essential layer for the correct visualization and quantification of the superficial and deep capillary plexus. To avoid the potential bias of misalignment in the inner retina, we only investigated perfusion of the full-thickness retina slab and choroidal sublayers in this study. All cases were only included after careful inspection of the segmentation.

When evaluating the choroid’s sublayers at baseline, no significant differences in CSC patients compared to the fellow control eyes could be detected. FRP, CCP, SLP, and HLP did not change over the four-week follow-up when evaluating all CSC patients after SML. As CSC is a condition of the pachychoroid disease spectrum, we also analyzed the ratio in perfusion between CC, SL, and HLP. It should be noted that, even though not statistically significant, the median of the perfusion ratio CCP/HLP was lower in the CSC group compared to fellow eyes. This indicates that these sublayers of the choroid might play a role in conditions of the pachychoroid disease spectrum. It has been shown that dilated vessels, or pachyvessels are predominantly found in the Haller’s layer in CSC [5] which may correspond to this finding. Over the follow-up period, perfusion values did not change significantly after SML in CSC patients. However, when looking at our subgroup analyses, we were able to detect several key findings. 

Group 1 consists of CSC patients without CNV in FLA or OCTA and had only received conservative treatment modalities in the past, e.g., lifestyle changes, acetazolamide, or eplerenone. Group 2 included PNV patients, most of whom received anti-VEGF-injections in the past. None of the patients in group 1 or 2 had received PDT or conventional laser treatments before inclusion.

BCVA did not show an increase after four weeks in both groups (*p* = 0.66 in group 1; *p* = 0.366 in group 2). The median CRT decreased in both groups, but statistical significance was reached only in group 2 (*p* = 0.594 in group 1; *p* = 0.009 in group 2). Interestingly, we detected a significant reduction in SFCT in the CSC group (*p* = 0.044), but not in the PNV group. The underlying mechanisms for the decrease in choroidal thickness induced by SML cannot be explained yet. Our SML protocol is adjusted to be able to irradiate RPE cells at an energy level below the threshold of cell death, which might lead to the rebuilding of the degraded RPE tight junctions, increasing pump function, and resulting in a decrease in subretinal fluid. Although it is conceivable that changes in the choroidal circulation may occur due to the effects of cytokines secreted into the basolateral direction by heated RPE cells, there is so far no basic evidence, and thus verification is necessary in future studies.

Different from the cases of CSC without CNV, the choroidal thickness of the PNV patients did not change after SML, although the retinal thickness was significantly decreased. In patients with PNV, there was no change in choroidal thickness, even though subretinal fluid was significantly reduced. Before SML, the choroidal thickness was increased in both groups, and there were no significant group differences, suggesting that CSC and PNV eyes respond differently to treatment with SML.

Subretinal fluid was also reduced in the PNV group, suggesting that the tight junctions and pump function of the RPE may have improved through RPE hyperthermia, but the effect of laser treatment on the choroidal side may have been masked for some reason, leading to a more moderate reduction in choroidal thickness. One reason for this might be the involvement of VEGF on the choroidal side. 

One limitation of the current study is the short follow-up period, even though effects are known to take several months to manifest. As described above, we aimed to gain deeper insights into short-term morphological and functional changes in chorioretinal perfusion through SML, so we focused on the results after 4 weeks. Furthermore, our treatment protocol allowed re-SML at each visit, which would have led to decreased subgroup sizes in later follow-ups. In our cohort, no one was lost to follow-up. Nevertheless, it is also necessary to investigate the effects on perfusion parameters over a longer period, such as after 3 months, 6 months, and 1 year, and for this purpose, a longer-term prospective control study is considered necessary. One of the present study’s limitations is the limited number of patients; thus, the statistical power is limited, and we had difficulty identifying smaller differences between groups. Furthermore, we examined a heterogeneous group of patients (ranging from 3 to 100 months disease duration), which especially hampers the detection of clinical and functional differences in subgroups. However, for our primary endpoint, in terms of perfusion changes in OCTA before and after SML, this limitation should not play an important role.

## 5. Conclusions

In summary, our data may provide several important insights into the pathophysiology of choroidal disease and the corresponding impact of SML. The results of this study show that microsecond pulsed laser therapy may affect not only the amount of subretinal fluid but also the choroidal thickness of CSC, and that different basic pathologies respond differently to the laser therapy. These results will be of great help in determining future treatment strategies for CSC. Further research is needed to clarify the mechanism of action and long-term outcome.

## Figures and Tables

**Table 1:** Demographic and clinical characteristics of enrolled CSC patients and control.

Parameter	CSC Eyes	Fellow Eyes
Gender (male/female), n (%)	17 (63.0)/10 (37.0)	11 (64.7)/6 (53.3)
Age (years)	54 (48.0; 63.0)	54 (49.5; 64.5)
Disease duration, months	20 (7; 48)	-
Previous treatment (conservative/Anti-VEGF), n (%)	15 (55.6)/12 (44.4)	-

Data are presented as median and quartiles (Q1; Q3). CSC: Central serous chorioretinopathy; SD: standard deviation; VEGF: vascular endothelial growth factor.

**Table 2:** Anatomical and functional parameters of CSC and control eyes at baseline examination.

Parameter	CSC Eyes	Fellow Eyes	*p*-Value
BCVA (logMAR)	0.4 (0.1; 0.14)	0.0 (0.0; 0.0)	**<0.001**
CRT (µm)	306 (254; 389)	271 (246; 295)	**0.047**
TMV (mm³)	8.7 (8.2; 9.4)	8,65 (8.3; 9.08)	0.697
SFCT (µm)	310 (286; 356)	275 (237; 312.8)	**0.022**
FRP (%)	26.44 (23.61; 29.52)	28.77 (23.49; 30.14)	0.540
CCP (%)	43.12 (38.79; 44.79)	46.35 (42.23; 47.08)	0.159
SLP (%)	60.43 (58.37; 63.11)	58.19 (57.04; 60.36)	0.126
HLP (%)	64.27 (61.24; 67.08)	61.88 (58.36; 67.33)	0.391
CCP/HLP	0.68 (0.58; 0.71)	0.74 (0.65; 0.79)	0.142
SLP/HLP	0.95 (0.92; 0.98)	0.93 (0.91; 0.99)	0.742

Comparison of all included CSC patients with unaffected fellow eyes at baseline examination. Mann–Whitney U test; all data are presented as median and quartiles (Q1; Q3); BCVA: best corrected visual acuity; CCP: choriocapillaris perfusion; CRT: central retinal thickness; CSC: Central serous chorioretinopathy; FRP: full retinal perfusion; HLP: Haller’s layer perfusion; SFCT: subfoveal choroidal thickness; SLP: Sattler’s layer perfusion; TMV: total macular volume.

**Table 3:** Comparison of the anatomical and functional parameters before and 4 weeks after SML for all treated patients.

Parameter	Baseline	4 Weeks	*p*-Value
BCVA (logMAR)	0.4 (0.1; 0.14)	0.3 (0.1; 0.5)	0.348
CRT (µm)	306 (254; 389)	266 (236; 324)	**0.024**
TMV (mm^3^)	8.7 (8.2; 9.4)	8.5 (7.9; 9.0)	0.62
SFCT (µm)	310 (286; 356)	299 (273; 337)	0.135
aSRF (µm^2^) SRF area (µm^2^)	0.17 (0.07; 0.43) 5.4 (1.4; 8.25)	0.075 (0.05; 0.33) 1.8 (0.7; 7.6)	0.115 0.123
LRT (µm)	364 (317; 465)	333 (272; 391)	**0.044**
CCP (%)	43.12 (38.79; 44.79)	42.35 (40.97; 43.94)	0.918
SLP (%)	60.43 (58.37; 63.11)	61.61 (59.9; 62.81)	0.469
HLP (%)	64.27 (61.24; 67.08)	63.63 (60.99; 66.71)	0.642
CCP/HLP	0.68 (0.58; 0.71)	0.65 (0.61; 0.71)	0.877
SLP/HLP	0.95 (0.92; 0.98)	0.95 (0.92; 1.01)	0.215
Lesional CCP (%)	20.45 (13.58; 38.39)	29.84 (17.06; 35,94)	0.352
Lesional SLP (%)	69.1 (57.57; 80.39)	65.33 (55.95; 74.0)	0.352
Lesional HLP (%)	72.58 (57.6; 82.8)	70.97 (53.96; 78.83)	0.352
Lesional CCP/HLP	0.29 (0.17; 0.61)	0.41 (0.24; 0.68)	0.326
Lesional SLP/HLP	0.98 (0.88; 1.02)	0.96 (0.83; 1.15)	0.959

Baseline and follow-up data in all included CSC patients. Wilcoxon signed rank test; all data are presented as median and quartiles (Q1; Q3). aSRF: apical subretinal fluid; BCVA: best corrected visual acuity; CCP: choriocapillaris perfusion; CRT: central retinal thickness; CSC: Central serous chorioretinopathy; FRP: full retinal perfusion; HLP: Haller’s layer perfusion; LRT: Lesional retinal thickness; SML: subthreshold microsecond pulsed laser; SFCT: subfoveal choroidal thickness; SLP: Sattler’s layer perfusion; TMV: total macular volume.

**Table 4:** Comparison of demographic parameters of patients with CSC without CNV and with CNV (PNV).

Parameter	CSC without CNV	PNV
Gender (male/female), n (%)	9 (64.3); 5 (35.7)	8 (61.5); 5 (38.5)
Age (years)	52 (45.75; 55.75)	60 (52.5; 65)
Disease duration, months	15 (7; 32.5)	36 (9; 78)

Demographic data of the subgroups. Data are presented as median and quartiles (Q1; Q3). CNV: choroidal neovascularization; CSC: Central serous chorioretinopathy; PNV: pachychoroid neovasculopathy; SD: standard deviation; VEGF: vascular endothelial growth factor.

**Table 5:** Change in anatomical and functional parameters four weeks after SML in each subgroup.

Parameter	CSC without CNV			PNV		
	Before SML	After SML	*p*-Value	Before SML	After SML	*p*-Value
BCVA (logMAR)	0.1 (0.1; 0.4)	0.15 (0.075; 0.4)	0.660	0.4 (0.35; 0.6)	0.4 (0.3; 0.55)	0.366
CRT (µm)	279 (246; 385)	260 (245; 326)	0.594	325 (279; 411)	267 (211; 325)	**0.009**
TMV	8.45 (7.9; 9.73)	8.25 (7.88; 9.53)	0.365	8.8 (8.35; 9.15)	8.6 (7.95; 8.9)	0.090
SFCT (µm)	329 (295; 373.75)	299 (284; 352)	**0.044**	299 (265; 332)	301 (272; 337)	0.929
aSRF (µm^2^) SRF area (µm^2^)	0.12 (0.06; 0.55) 4.2 (1.35; 7.65)	0.065 (0.033; 0.196) 1.4 (0.8; 6.33)	0.208 0.208	0.26 (0.09; 0.39) 5.9 (1.55; 9.38)	0.17 (0.05; 0.41) 5.05 (0.375; 7.75)	0.326 0.406
LRT (µm)	370.5 (310.5; 499.5)	347 (274.25; 405.75)	0.300	364 (310; 426.5)	326 (240.5; 405)	0.055
FRP (%)	25.85 (23.27; 28.39)	24.88 (22.75; 28.43)	0.646	26.44 (23.91; 31.02)	24.91 (23.37; 29.12)	0.753
CCP (%)	43.39 (38.32; 46.51)	42.54 (40.9; 44.47)	0.959	41.92 (39.26; 44.48)	41.77 (40.27; 44.23)	0.600
SLP (%)	60.43 (58.02; 63.16)	61.88 (59.66; 62.86)	0.575	60.86 (58.66; 63.57)	61.22 (59.99; 63.47)	0.600
HLP (%)	64.19 (60.66; 68.01)	64.27 (60.63; 67.39)	0.878	65.02 (61.28; 66.57)	63.14 (61.15; 65.91)	0.463
CCP/HLP	0.68 (0.57; 0.74)	0.66 (0.61; 0.71)	0.878	0.65 (0.59; 0.71)	0.66 (0.60; 0.74)	0.463
SLP/HLP	0.95 (0.92; 0.97)	0.95 (0.92; 0.98)	0.575	0.95 (0.91; 0.99)	0.97 (0.92; 1.0)	0.075
Lesion CCP (%)	22.44 (18.55; 42.67)	30.77 (19.51; 33.97)	0.799	12.86 (10.11; 38.2)	24.85 (10.14; 49.78)	0.294
Lesion SLP (%)	67.47 (56.65; 75.99)	64.37 (59.68; 71.06)	0.445	74.25 (55.47; 86.53)	73.07 (48.67; 81.99)	0.463
Lesion HLP (%)	73.39 (58.46; 82.55)	73.64 (52.19; 81.38)	0.575	72.58 (50.66; 86.01)	69.34 (51.53; 80.15)	0.249
Lesion CCP/HLP	0.29 (0.27; 0.71)	0.41 (0.32; 0.54)	0.799	0.18 (0.13; 0.65)	0.46 (0.13; 0.94)	0.249
Lesion SLP/HLP	0.99 (0.84; 1.0)	0.95 (0.79; 1.11)	0.799	0.96 (0.91; 1.14)	1.04 (0.88; 1.27)	0.917

Subgroup analyses at baseline compared to the follow-up visit. Wilcoxon signed rank test, all data are presented as median and quartiles (Q1; Q3). Conservative: group of patients without prior injections or photodynamic therapy. Anti-VEGF: group of patients with prior Anti-VEGF injections; BCVA: best corrected visual acuity; CCP: choriocapillaris perfusion; CNV: choroidal neovascularization; CRT: central retinal thickness; CSC: Central serous chorioretinopathy; FRP: full retinal perfusion; HLP: Haller’s layer perfusion; LRT: Lesional retinal thickness; PNV: pachychoroid neovasculopathy; SML: subthreshold microsecond pulsed laser; SFCT: subfoveal choroidal thickness; SLP: Sattler’s layer perfusion; TMV: total macular volume.

**Table 6:** Comparison of anatomical and functional parameters of the two subgroups before and four weeks after SML.

Parameter	Before SML			After SML		
	CSC without CNV	PNV	*p*-Value	CSC without CNV	PNV	*p*-Value
BCVA (logMAR)	0.1 (0.1; 0.4)	0.4 (0.35; 0.6)	**0.007**	0.15 (0.075; 0.4)	0.4 (0.3; 0.55)	**0.014**
CRT (µm)	278 (246; 385)	325 (279; 411)	0.259	260 (244.75; 325.75)	267 (213; 325	0.793
TMV (mm^3^)	8.45 (7.9; 9.7)	8.8 (8.35; 9.15)	0.519	8.25 (7.88; 9.53)	8.6 (7.95; 8.9)	0.685
SFCT (µm)	329 (295; 373)	299 (265; 333)	0.141	299 (284; 352)	301 (273; 337)	0.720
aSRF (µm^2^)	0.12 (0.06; 0.51)	0.26 (0.09; 0.39)	0.705	0.07 (0.033; 0.19)	0.17 (0.05; 0.41)	0.274
SRF area (µm^2^)	4.2 (1.35; 7.65)	5.9 (1.55; 9.38)	0.689	1.4 (0.8; 6.35)	5.05 (0.375; 7.57)	0.852
LRT (µm)	371 (311; 500)	364 (310; 427)	0.981	347 (274.25; 405.75)	326 (241; 405)	0.583
FRP (%)	25.85 (23.26; 28.39)	26.44 (23.91; 31.02)	0.635	24.88 (22.75; 28.43)	24.91 (23.37; 29.12)	0.792
CCP (%)	43.39 (38.32; 46.51)	41.92 (39.26; 44.48)	0.792	42.54 (40.92; 44.47)	41.77 (40.27; 44.23)	0.562
SLP (%)	60.43 (58.02; 63.17)	60.86 (58.66; 63.57)	0.792	61.89 (59.66; 62.86)	61.22 (59.99; 63.47)	0.958
HLP (%)	64.19 (60.66; 68.01)	65.02 (61.28; 66.56)	0.958	64.27 (60.63; 67.39)	63.14 (61.15; 65.91)	0.562
CCP/HLP	0.68 (0.57; 0.74)	0.65 (0.59; 0.71)	0.875	0.66 (0.61; 0.71)	0.66 (0.60; 0.74)	0.958
SLP/HLP	0.95 (0.92; 0.99)	0.95 (0.92; 0.99)	0.958	0.95 (0.92; 0.98)	0.97 (0.92; 1.02)	0.713
Lesion CCP (%)	22.44 (18.55; 42.67)	12.85 (10.11; 38.20)	0.118	30.77 (19.51; 33.97)	24.85 (10.14; 49.78)	0.635
Lesion SLP (%)	67.47 (56.65; 75.99)	74.25 (55.47; 86.53)	0.428	64.37 (59.68; 71.06)	73.07 (48.67; 81.99)	0.428
Lesion HLP (%)	73.39 (58.46; 82.55)	72.58 (58.46; 82.55)	0.958	73.0 (52.19; 81.38)	69.34 (51.53; 80.15)	0.713
Lesion CCP/HLP	0.29 (0.27; 0.72)	0.18 (0.13; 0.65)	0.220	0.41 (0.32; 0.54)	0.46 (0.13; 0.95)	1.0
Lesion SLP/HLP	0.99 (1.03; 0.85)	0.96 (0.91; 1.16)	1.000	0.95 (0.79; 1.11)	1.04 (0.88; 1.27)	0.368

Comparing the subgroups with CSC and PNV. Mann–Whitney U test; all data are presented as median and quartiles (Q1; Q3). aSRF: apical subretinal fluid; BCVA: best corrected visual acuity; CCP: choriocapillaris perfusion; CNV: Choroidal neovascularization; CRT: central retinal thickness; CSC: Central serous chorioretinopathy; FRP: full retinal perfusion; HLP: Haller’s layer perfusion; PNV: pachychoroid neovasculopathy; SFCT: subfoveal choroidal thickness; SLP: Sattler’s layer perfusion; SML: subthreshold microsecond pulsed laser; TMV: total macular volume.

## Data Availability

The data presented in this study are available on request from the corresponding author. The data are not publicly available.

## Abbreviations

aSRF	apical subretinal fluid
BCVA	best corrected visual acuity
CC	choriocapillaris
CCP	choriocapillaris perfusion
CNV	choroidal neovascularization
CSC	central serous chorioretinopathy
EDI	enhanced depth of imaging
EDTRS	Early Treatment Diabetic Retinopathy Study
FA	fluorescein angiography
FAF	fundus autofluorescence
FR	full retina
FRP	full retina perfusion
HL	Haller’s layer
HLP	Haller’s layer perfusion
ICGA	indocyanine green angiography
LRT	lesional retinal thickness
OCT	optical coherence tomography
OCTA	optical coherence tomography angiography
PDT	photodynamic therapy
PNV	pachychoroid neovasculopathy
RPE	retinal pigment epithelium
SFCT	subfoveal choroidal thickness
SML	subthreshold micropulse laser
SL	Sattler’s layer
SLP	Sattler’s layer perfusion
VEGF	vascular endothelial growth factor
SRF	subretinal fluid
TMV	total macular volume
